# The Royal Free Hospital and the War

**Published:** 1916-06-17

**Authors:** 


### THE TREATMENT OF WOUNDED OFFICERS.

### The Royal Free Hospital and the War.

Besides providing ward accommodation for naval and military patients, this hospital has to its credit the organisation of a unit for Serbia and the equipment and management of a large hospital for officers. In addition to these activities the hospital has been called upon to train a large and growing number of women medical students, who are preparing to fill the gaps in the ranks of practitioners which the war has caused. When it is remembered that all such students who receive their training in London are trained at the Royal Free Hospital some idea can be obtained of the extent to which the influence of the war has been felt in this connection alone. But important as this work is, and far-reaching as its effect may be on the future of the profession, it is by no means the most striking of the duties which this hospital has volunteered or been called upon to perform.

### The New Out-Patients' Block.

Shortly after the outbreak of war forty beds were allotted to naval and military cases, and for a long period these have been in constant occupation. As soon as the new out-patients' department was complete the whole block was offered to the War Office and accepted for use as a hospital for officers. This block, the building of which was begun in 1912, is erected on a piece of land immediately adjoining the hospital at the rear, the freehold of which was secured for £14,000. Had the conditions been normal this block, known as the Helena Building, would now have been in use as an out-patients' and maternity department; but as the war broke out before it was ready to serve the purpose for which it was built, and as the needs of the military authorities were urgent, it was decided to remain in the old quarters for the time being and to place the new block at the disposal of the Government as a hospital for officers, containing 140 beds.

### A Hospital for Officers.

The new block is a three-storeyed building which, when it is no longer required for the purpose for which it has been temporarily adapted, will add enormously to the value of the hospital. The ground floor of the building contains in the centre what will in due course be used as the out-patients' hall; this is a spacious apartment of about 80 ft. by 30 ft., which has been transformed into a commodious and airy ward. On the left of the entrance from the main building one passes through the billiard-room, the table and all the furniture in which were presented by a generous donor. The physicians' rooms, or rather what will be the physicians' rooms when the building is no longer required as a war hospital, are situated on the north side, and the dispensary, almoner's office, and surgeons' rooms on the south side. These are now for the most part furnished as small wards, one or two of them containing single beds only and others two or more, several of them being sufficiently large for six or eight beds. Similarly, the ground floor of the casualty block, which stands at the north-east corner of the main new building, is furnished as to several apartments as wards. The second floor was planned to make provision for the dental, massage, *x*-ray, and electrical departments, students' quarters, and a suite of rooms for the nursing staff, the latter connected by a private staircase with a similar suite on the floor above.

Some of these apartments are equipped for the purpose for which they were originally designed, thus an *x*-ray apparatus has been installed in its proper quarters, and the massage-rooms are also furnished and used for the treatment of officers requiring massage. One of the larger rooms on this floor is used as the officers' dining-room. Extending over the casualty block on this floor are rooms which, when the building reverts to the use for which it was intended, will be allotted to the resident domestic staff. The whole of the second floor, with the exception of the nurses' rooms, was intended for the purpose of a complete maternity department, comprising one ward of eight beds, a labour ward with three, and an operating theatre, while connected with the department is a residential portion which, when the War Office has no further need of it, will be allotted to the obstetric staff.

All the furniture and equipment, except such as were the gift of generous helpers, were provided by the War Office. The furnishing of the wards and the recreation and dining rooms has been done remarkably well, and it is obvious that those who were responsible for the selection of the furniture had in mind the desirability of making the rooms as cheerful and comfortable as it is possible for a hospital to be. The single-bed wards to which reference has just been made are not, as might be imagined, reserved for officers of higher rank, but an officer for whom absolute quiet is essential is placed in one of these wards, or if an officer expresses a preference for a private room and one is vacant he is allowed to have it. But there is no hard-and-fast rule, and two generals have occupied beds in the large ward on the ground floor.

This hospital for officers is controlled entirely by the weekly board of the Royal Free Hospital, but the pay of the salaried staff is provided by War Office funds, and the maintenance of the officers' hospital does not encroach in any way on the funds of the hospital. Forty R.A.M.C. orderlies are on duty in the military sections as well as a number of Army Nursing Sisters. Mr. Reginald R. Garratt, the secretary of the Royal Free Hospital, is honorary officer in charge of the military section, having full authority over it; and Miss R. Cox-Davies, the matron, is hon. matron of the officers' hospital. It is not without interest to note that the Royal Free Hospital has an hereditary association with the Army, the site it now occupies having been taken over in 1842 from the Light Horse Volunteers, whose barracks stood there.

